# The Principles of the Radical Cure of Inguinal Hernia1Read at the thirty-first annual meeting of the Medical Association of Central New York, at Auburn, October 18, 1898

**Published:** 1899-01

**Authors:** William B. Jones

**Affiliations:** Rochester, N. Y.


### THE PRINCIPLES OF THE RADICAL CURE OF INGUINAL HERNIA.[1]

By WILLIAM B. JONES, M. D., Rochester, N. Y.

THE internal inguinal ring is at the apex of a funnel-shaped depression in the anterior abdominal wall that concentrates intra-abdominal pressure at that point. The inguinal canal begins there and passes outward and downward obliquely through the muscles and fasciæ so that pressure directly outward from within tends to close its walls more firmly together. It is weakest toward force exerted in its own direction obliquely. Its walls are cemented together by cellular tissue, through which pass the cord and spermatic vessels, and they may vary in size according to their distention. When collapsed as from pressure the descent of hernia is slightly favored.

In operating after dividing skin and fat and treating the sac, the canal must be firmly closed in one of two ways. Bassini's method restores the two layers, the posterior being transversalis fascia, and transversalis and internal oblique muscles with their aponeuroses all stitched to Poupart's ligament, the internal ring at the upper end of the seam just large enough for passage of cord and vessels without constriction, and with one stitch above them to prevent subsequent splitting of the muscle and a new hernia higher up than the old one. The anterior wall is external oblique muscle and aponeurosis laid over the cord and joined also to Poupart's ligament, the external ring at its lower end, covered only by fat and skin. If primary union be secured and the sutures remain until it is strong, the method is very successful, although it has the original tendency, due to being at the apex of the infundibulum of the anterior abdominal wall and occupied by vessels of varying caliber.

This is overcome by Halsted's method of separating the fibers of all the muscles farther upward and outward, till the outer end of the funnel is reached, placing the cord and its vessels away up there, bringing them through the entire thickness of the muscles and then closing the canal firmly, uniting its margins from end to end ; not in two layers to restore the canal, but in one to obliterate it, being careful as before, to avoid constriction of the cord, and to have it emerge between two sutures, not above the uppermost one. The skin is then reunited ; closure gathers in enough tissue to nearly or

---

1. Read at the thirty-first annual meeting of the Medical Association of Central New York, at Auburn, October 18, 1898

quite level up the depression. Now there is no infundibulum to promote hernia, and no internal ring where hernia usually starts, so it doesn't start; no canal, no external ring.

Wherever the cord structures emerge, there must be an opening just large enough to admit them. In aponeurotic tissue, as at the internal ring, it is proper to consider it an opening, because although fully occupied when the vessels are filled, a very little pressure empties them and leaves room for an entering wedge. Not so in muscular tissue; the veiy cause of such pressure is contraction of those same muscles, whereby those fibers that surround the opening thicken and broaden, themselves first occupying any space there created.

There are only two other methods worth considering: In one the sac instead of being cut off is crumpled into a mass and replaced just inside the internal ring, forming a mound opposing the constant pressure there concentrated. In the other a separate opening is made in the abdomen, about on a level with the spine of the ileum. A long needle and suture, or a pair of forceps are passed through it into the peritoneal cavity down the inguinal canal. The sac is seized and drawn up out of this opening as far as may be, stitched fast and cut off. The canal is then restored as in Bassini's operation, the cord traversing it as before.

The only reliable material for suturing the margins of the canal is silver wire. If properly placed it nearly always remains permanently without inconvenience. Some day we shall have something that can be depended upon to remain just long enough to secure firm union and then be absorbed, but there is none yet.

Primary union is practically essential. Scar tissue formed by granulation is yielding and usually stretches so that trouble recurs. All the methods depending upon the open treatment have been discarded for that reason. In the operations I have advocated if stitch abscess occurs it often defeats the attempt at cure. They should not occur.

After operation the pressure of a truss is unnecessary and would cause irritation over the buried sutures and absorption and weakening of the cicatrix.

A few words about results. In 610 operations by DeGarmo and Coley there were patients of all ages from five months to eighty years, some having appendix, cecum, ovary, or intraperitoneal tumor, besides usual contents. Many were irreducible, quite a number acutely strangulated. Of all these, only one died, and he of pneumonia. Sixteen were not cured by the first operation, but about half of them were by a second, leaving about one out of one hundred not cured.

The argument is unanswerable for operation of all hernia, not cured by faithful use of truss for a year or so. I would never wear one myself and would be operated upon at the first appearance of hernia. The discomfort, expense and danger of a radical cure are all less than from enduring a truss for a life-time.

SUMMARY.

The organic causes of hernia is a broad infundibulum, with its apex at the entrance of the inguinal canal, and the weakness of that part of the abdominal wall made still weaker when the vessels in the canal are collapsed. The indications in radical treatment are to level up that depression as much as possible, transplant the cord to a place not affected by that formation, obliterate the inguinal canal by suturing, secure complete primary union of all layers. Place the uppermost suture above the upper end of muscle separation. Until reliable absorbable material is to be had use silver wire as a buried permanent suture. Never put on a truss again.

Dr. Buchanan, of Pittsburg, says: "When we consider the high percentage of cures and practical freedom from danger in these operations; that a patient may in two weeks, without pain and with little inconvenience, be freed from the danger of strangulation, the burden of a truss and the constant sense of insecurity—when we consider these facts, it is incomprehensible that all physicians do not advise the radical cure for all their patients, except those rare cases where, for special reasons, operation is contraindicated."

215 LAKE AVENUE.

